# The psychoactive cathinone derivative pyrovalerone alters locomotor activity and decreases dopamine receptor expression in zebrafish (*Danio rerio*)

**DOI:** 10.1002/brb3.1420

**Published:** 2019-10-18

**Authors:** Christopher Laurence Souders, Robert H. Davis, Hua Qing, Xuefang Liang, Marcelo Febo, Christopher J. Martyniuk

**Affiliations:** ^1^ Department of Physiological Sciences and Center for Environmental and Human Toxicology University of Florida Genetics Institute Interdisciplinary Program in Biomedical Sciences Neuroscience College of Veterinary Medicine University of Florida Gainesville FL USA; ^2^ Inner Mongolia Key Laboratory of Environmental Pollution Control & Waste Resource Reuse School of Ecology and Environment Inner Mongolia University Hohhot China; ^3^ Department of Psychiatry Evelyn F. and William L. McKnight Brain Institute College of Medicine University of Florida Gainesville FL USA

**Keywords:** bath salts, behavioral screening, drug abuse, high‐throughput, MDPV

## Abstract

**Introduction:**

Pyrovalerone (4‐methyl‐β‐keto‐prolintane) is a synthetic cathinone (beta‐keto‐amphetamine) derivative. Cathinones are a concern as drugs of abuse, as related street drugs such as methylenedioxypyrovalerone have garnered significant attention. The primary mechanism of action of cathinones is to inhibit reuptake transporters (dopamine and norepinephrine) in reward centers of the central nervous system.

**Methods:**

We measured bioenergetic, behavioral, and molecular responses to pyrovalerone (nM‐µM) in zebrafish to evaluate its potential for neurotoxicity and neurological impairment.

**Results:**

Pyrovalerone did not induce any mortality in zebrafish larvae over a 3‐ and 24‐hr period; however, seizures were prevalent at the highest dose tested (100 µM). Oxidative phosphorylation was not affected in the embryos, and there was no change in *superoxide dismutase 1* expression. Following a 3‐hr treatment to pyrovalerone (1–100 µM), larval zebrafish (6d) showed a dose‐dependent decrease (70%–90%) in total distance moved in a visual motor response (VMR) test. We interrogated potential mechanisms related to the hypoactivity, focusing on the expression of dopamine‐related transcripts as cathinones can modulate the dopamine system. Pyrovalerone decreased the expression levels of *dopamine receptor D1* (~60%) in larval zebrafish but did not affect the expression of *tyrosine hydroxylase*, *dopamine active transporter*, or any other dopamine receptor subunit examined, suggesting that pyrovalerone may regulate the expression of dopamine receptors in a specific manner.

**Discussion:**

Further studies using zebrafish are expected to reveal new insight into molecular mechanisms and behavioral responses to cathinone derivates, and zebrafish may be a useful model for understanding the relationship between the dopamine system and bath salts.

## Graphical abstract

Hypoactivity is induced by pyrovalerone in zebrafish larvae.

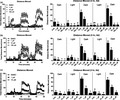

## 1 INTRODUCTION

Cathinone ((S)‐2‐amino‐1‐phenyl‐1‐propanone) is a beta‐ketone amphetamine analog produced by the plant *Catha edulis* (Khat), a species endemic to the Horn of Africa and Arabia. Cathinone derivatives, often referred to as “bath salts,” encompass different chemical moieties that determine their level of selectivity and affinity for specific monoamine neurotransmitter transporters in the central nervous system (CNS), such as dopamine (SLC6A3, DAT) and norepinephrine transporter (SLC6A2, NET) (Cameron, Kolanos, Solis, Glennon, & De Felice, 2013). Dopaminergic modulation in the CNS by cathinones is thought to be a major factor underlying the reasons why these compounds are sought after as recreational drugs, as they induce euphoria, increase libido, and increase energy. However, cathinones have also been associated with cardiotoxicity and neurotoxicity. In humans, components of bath salts can induce cardiac abnormalities (e.g., arrhythmias, hypertension, tachycardia) as well as psychiatric and neurological sequelae (e.g., ataxia, aggression, confusion, hallucination; Prosser & Nelson, 2012). Deeper investigations into the diversity of biological and behavioral responses are warranted to define the full scope of cathinone‐induced neurotoxicity.

Cathinone derivatives include pyrovalerone, methylenedioxypyrovalerone (MDPV), butylone, methylone, and α‐pyrrolidinopentiophenone (i.e., “Flakka”). These chemicals are sold illegally as “bath salts” which have become increasingly problematic for drug use disorders. MDPV, for example, can be highly addictive and can induce psychostimulant‐like effects that can be a magnitude more potent than cocaine and 10 times more powerful and longer lasting (Baumann et al., 2013). Whereas MDPV is arguably one of the more widely studied cathinones, virtually little is known about new emerging analogs. Noteworthy is that cathinones exhibit pharmacological variability in potency and can affect solute transporters differently (Simmler et al., 2013), resulting in complex physiological and behavioral responses. Therefore, mechanistic and behavioral studies that address the complexity and toxicity of a range of cathinones are needed to better understand their potential for abuse.

Cathinones and their derivatives can lead to neurotoxicity, and studies demonstrate that dopamine‐synthesizing cells are a significant target. Acute toxicity of cathinones at the cellular level includes the production of reactive oxygen species, oxidative damage, and cytotoxicity. In human SH‐SY5Y, a neuroblastoma cell line used in neurotoxicity studies, Valente et al. (2016) showed that both methylone and MDPV induced autophagy. In addition, Den Hollander et al. (2015) treated SH‐SY5Y cells with 4‐methylmethcathinone (4‐MMC) and 3,4‐methylenedioxymethcathinone (MDMC) and showed that cathinone derivates decreased mitochondrial respiration and oxidative phosphorylation in cells. These studies point toward mitochondrial dysfunction as a potential mechanism of toxicity.

Zebrafish are a widely used neurobehavioral model for high‐throughput screening of environmental toxins, pharmaceuticals, and illicit drugs. There is conservation of neurological pathways that control behavior between fish and mammals (Kalueff, Stewart, & Gerlai, 2014; Panula et al., 2010); thus, the zebrafish model is amendable to high‐throughput screening to discern the effects of drugs. Zebrafish have been used to study the behavioral effects associated with cocaine (Darland & Dowling, 2001; López‐Patiño, Yu, Cabral, & Zhdanova, 2008), heroin and cannabis (Stewart & Kalueff, 2014), and legal stimulants such as caffeine (Ladu, Mwaffo, Li, Macrì, & Porfiri, 2015). More recently, zebrafish as a model has been proposed to investigate α‐pyrrolidinopentiophenone (i.e., “Flakka”), a synthetic stimulant of the cathinone class (Kolesnikova, Khatsko, Demin, Shevyrin, & Kalueff, 2018). A range of physiological and behavioral endpoints can be measured in zebrafish that are relevant for drug‐taking behaviors, such as motor activity (e.g., locomotion and distance traveled), withdrawal syndrome (Cachat et al., 2010), and anxiolytic/anti‐anxiolytic behaviors (Chakravarty et al., 2013). We posit that zebrafish can also be a useful behavioral screen for elucidating cathinone‐induced neuroadaptations to better understand drug abuse behaviors.

In this study, pyrovalerone was investigated as a test drug to determine the applicability of the zebrafish model for behavioral and molecular screening of synthetic cathinones. Zebrafish embryos can act as intact whole animal sensors for screening effects on the mitochondrial bioenergetic phenotype (Wang, Souders, Zhao, & Martyniuk, 2018a, 2018b). We also measured different larval behaviors (i.e., locomotor activity, anxiolytic behaviors) following treatment with pyrovalerone, as other drugs of abuse have been reported to induce both hyper‐ and hypoactivity in zebrafish. We also measured the expression of the dopaminergic system in larvae, as cathinones modulate the dopamine system. We hypothesized that zebrafish treated with pyrovalerone would exhibit impaired oxidative respiration and induction of superoxide dismutase 1. We also hypothesized that pyrovalerone would induce hyperactivity in larvae and promote anxiolytic‐like behaviors.

## 2 MATERIAL AND METHODS

### 2.1 Zebrafish breeding

All animal experiments were approved by the University of Florida Institutional Animal Care and Use Committee and conformed to NIH guidelines for animal use and euthanasia. Adult male and female zebrafish (ABTu strain) were housed in the Cancer Genetics Research Center (Animal Care Services Facility, University of Florida) in a Pentair Aquatic Eco‐systems Z‐Mod stand‐alone recirculating system (Pentair). Temperature and pH were monitored daily (mean water pH was 7.2 ± 1, and mean temperature was 27.4 ± 1°C). Dissolved oxygen was ~6.6 ppm, and this was measured using a LaMotte^®^ Freshwater Fish Farm test kit. Fish were subjected to 14‐hr light and 10‐hr dark daily cycle.

For breeding, zebrafish were randomly selected from a breeding stock and placed in a shallow water breeding tank the night before embryo collection. Two males to two females were placed into a single tank, and two tanks were set up to generate fertilized embryos for experiments. Multiple experiments were conducted for behavior, and eggs were derived from randomized parents (derived from 5 or 6 different males and females) to minimize clutch effects for behavior. A divider separated the males and females overnight. These dividers were removed at 8:00 a.m. when the facility lights turned on and spawning occurred. Embryos were pooled into three petri dishes, totalling ~450 embryos. Using a light microscope, unfertilized eggs were identified and removed. Fertilized eggs were equally distributed into eight petri dishes, and each dish contained ~50 embryos. Embryos were maintained in an incubator at 27°C ± 1.0°C and exposed to the same light‐to‐dark schedule as above. Behavioral assays following 3‐ and 24‐hr exposure were conducted two or three times independently for rigor.

### 2.2 Pyrovalerone preparation

Pyrovalerone (CAS # 1485 CV; RS‐1‐(4‐methylphenyl)‐2‐(1‐pyrrolidinyl)pentan‐1‐one hydrochloride; Lipomed, Inc.) was prepared to yield a 100 millimolar (mM) stock solution. Stock solutions were stored at −20°C, with working solutions prepared immediately prior to all embryo and larval exposure. Working solutions were prepared fresh on the day of each exposure for the different biological assays. The DMSO solvent control had a final concentration of 0.1%, as the effects of DMSO at this concentration on the physiology and development of zebrafish at 0.1% are considered negligible (Hallare, Nagel, Kohler, & Triebskorn, 2006). The full recipe for embryo rearing media can be found in the Zebrafish book (Westerfield, 2000) (https://zfin.org/zf_info/zfbook/chapt1/1.3.html). In human intoxications and drug overdoses, cathinones are detected in the blood or tissues at levels in the 10–500 ng/ml range (Marinetti & Antonides, 2013). The doses used in this study ranged from 245 ng/ml to 24.5 µg/ml (1–100 µM); thus, the lowest dose tested is physiologically relevant for drug users.

### 2.3 Metabolic capacity and oxidative phosphorylation state

For metabolic assays, embryonic zebrafish were exposed to pyrovalerone for 24 hr and oxygen consumption rate (OCR) was measured in a mitochondrial stress test. We hypothesized that pyrovalerone would affect oxidative respiration and mitochondrial bioenergetics. Larval zebrafish were exposed starting at ~6 hpf (hours postfertilization) until 30 hpf to pyrovalerone (DMSO or one dose of 1, 10, or 100 µM) in separate glass beakers (five beakers per treatment). Following the 24‐hr exposure, one embryo (*N* = 5 total) from each of the five biological replicates/treatment was selected for mitochondrial respiration assessment. The XFe24 Extracellular Flux Analyzer (Agilent) measures oxygen consumption and pH change over time using solid‐state sensor probes. Each well of an Islet Plate was filled with 1 ml of XF Calibrant fluid, and the Islet Plate was incubated with the sensor plate overnight at 28°C. Each well of an islet capture microplate contained an initial volume of 425 µl of embryo rearing media (ERM). After washing ERM twice, a single embryo was added to each islet well with 100 µl ERM (five wells per treatment). Thus, the total volume of ERM in each well was 525 µl. Blanks contained 525 µl ERM with no embryos (*N* = 4). The instrument was programmed to add a volume of 75 µl each of challenge solutions of oligomycin (75.2 μM), carbonyl cyanide‐p‐trifluoromethoxyphenylhydrazone (FCCP, 54 μM), and sodium azide (NaN_3_, 200 mM) to give final concentrations in the wells of 9.4, 6, and 20 mM, respectively. The protocol consisted of the following time cycles: 2 min for mixing, 1 min paused, and then 2 min to measure oxygen levels and pH. Ten cycles of data were collected for basal respiration. Eighteen cycles were used for oligomycin to inhibit ATP‐dependent respiration of embryos. Eight cycles were set for the FCCP incubations, which maximizes respiration. Sodium azide was introduced last for 24 cycles to completely inhibit mitochondrial respiration of zebrafish embryos.

### 2.4 Behavioral assays

#### 2.4.1 Visual motor response test

To assess behavioral responses in zebrafish larvae, we utilized a Visual motor response (VMR) test (in some cases as dark photokinesis or the white–dark challenge test) and measured total distance traveled (mm) over 1‐min bins (i.e., measure of larval activity). The most dramatic response expected in the test typically occurs in the last two cycles of dark (20–30 min and 40–50 min). Zebrafish larvae will become more active during the dark period as they seek the light (i.e., dark photokinesis) (Burgess & Granato, 2007; Fernandes et al., 2012). The test has been referred to in the literature as different tests, and herein we refer to this as the VMR test.

For the VMR test, zebrafish were first raised in beakers with 10 ml of ERM up to 5 dpf (days postfertilization) with daily water changes. At 5 dpf (126 ± 1 hpf) or 6 dpf (150 ± 1 hpf), larvae were transferred into 100 µl of ERM in a 96‐well plate (the plate was precoated 24 hr prior to the assay with pyrovalerone, washed three times with ERM, and allowed to dry on the day of the treatment). Zebrafish larvae were exposed directly in a round well plate to pyrovalerone. Zebrafish larvae were exposed to one dose of either DMSO or 1, 10, or 100 µM pyrovalerone for 3 or 24 hr for the behavioral assays. To prepare the exposure solution, 1000x solutions were prepared in 100% DMSO and then diluted 1/500 for 2x exposure stocks in ERM. Then, 100 µl of this 2x exposure solution in ERM was added to each well (containing 100 µl ERM and a single larva) using a multi‐pipette (i.e., 1/2 dilution of 2x stock). Assays were run in early afternoon, and the time was kept consistent; plates for the 3‐hr treatments were placed directly into a DanioVision™ instrument (Noldus) with temperature control unit set to 26°C ± 1. The assay start time was adjusted so that the behavior assay began directly at the 3‐hr mark. The plate containing zebrafish larvae for the 24‐hr experiment was placed into a 26°C ± 1 incubator until the next day.

Four independent experiments were conducted with 5 or 6 dpf larvae depending on the assay conducted. Treatments lasted for either 3 hr (Experiments 1–3) or 24 hr (Experiment 4). Once the 96‐well plate was transferred into the DanioVision™ instrument, it was situated in a warm, circulating water bath (27 ± 1°C). After treatment with pyrovalerone for 3 or 24 hr, the movement of fish was simultaneously and individually tracked using an infrared analog camera installed in the DanioVision™ Observation Chamber. Larvae were tracked following a standard 50‐min light routine: 10 min dark, 10 min white light, 10 min dark, 10 min white light, and 10 min dark. The camera was connected to a USB port on the EthoVision® XT (Noldus Information Technology) computer to digitize the analog signal.

Separate behavioral trials were performed (*N* = 16–20 fish/group in Experiment 1, *N* = 8–12 fish/group in Experiment 2, *N* = 12–16 fish/group in Experiment 3, and *N* = 10 fish/group in Experiment 4 for the 24‐hr experiment). Data for each of the five time periods were analyzed separately for distance moved (mean distance moved (mm)/min), and each experiment was analyzed independently. For each experiment, group data were binned into a mean for all fish every minute. In each of the five time intervals, there were 10 data points (mean distance) collected over 10 min that were compared among groups for total distance moved, which is a measure of activity. After the exposure period, surviving larvae (more than > 95% survival) from one beaker were pooled into a single tube. Samples were flash‐frozen using liquid nitrogen and stored at −80°C for RNA extraction.

#### 2.4.2 Light–dark preference test

To test anxiolytic and anti‐anxiolytic behavior, four separate behavioral trials were performed. Experiments 1 and 2 were 3‐hr exposures, and Experiments 3 and 4 were 24‐hr exposures (*N* = 24 fish/group in Experiment 1, *N* = 19–23 fish/group in Experiment 2, *N* = 19–24 fish/group in Experiment 3, and *N* = 23–24 fish/group in Experiment 4). Fish were raised as above for the VMR test until 5 dpf. Larval zebrafish were pipetted individually in 200 µl of ERM to a square 96‐well plate and exposed as above for 3 or 24 hr to pyrovalerone. Treatments were randomized across the 96‐well plate to reduce any bias for positional effects. Care was taken to avoid damaging and selecting nondeformed larvae. Plates were placed into a 26°C ± 1.0 incubator for 3 or 24 hr and then were transferred to a DanioVision Observation Chamber with temperature control unit set to 27 ± 1°C above a grid blocking light to half of each well while still allowing infrared detection by the camera. All assays were conducted in early afternoon. A cover of the same material was placed on top of the well plate to prevent reflected light from obscuring the dark zones. In EthoVision, zones were established for each well under the Analysis Profile and checked for accuracy using Detection Settings. This assay consists of an hour‐long video with light control set to 100% and a 0‐s fade duration with an additional 20‐min delay before recording was started (to acclimate the fish and ensure appropriate tracking). The activity analysis threshold was set to 16 in Detection Settings. This was the minimum threshold determined to track each larva appropriately without counting background noise as movement. After each recording, Track Visualization was used to screen for poorly tracked or dead embryos, and these were censored before data were analyzed. Analysis Profiles were generated in EthoVision and exported for further analysis in GraphPad PRISM.

### 2.5 RNA extraction and cDNA synthesis

Gene expression analysis was conducted in larvae (7 dpf) exposed to pyrovalerone for 24 hr (starting at 6 dpf). This corresponded to the 24‐hr exposure in the behavioral experiments. We reasoned that this longer time point would be needed for capturing expression changes in the dopamine system, compared to the shorter 3‐hr exposure. Zebrafish were exposed in glass beakers to solvent control or pyrovalerone at 1 and 10 µM (*N* = 8–10). Extraction of RNA from larvae pools was performed using 500 μl TRIzol^®^ reagent (Life Technologies) as per the manufacturer's protocol. Immediately after extraction, RNA pellets were dissolved in 100 µl of RNase–DNase‐free water and were purified via the RNeasy Mini Kit column, as per the manufacturer's protocol (Qiagen). An on‐column DNase treatment was performed to remove any genomic DNA (gDNA). Purified RNA samples were assessed for quality using the 2100 Bioanalyzer (Agilent Technologies). The mean RIN value for RNA was 8.70 (*SD* ± 1.16). The concentration of RNA was determined using the NanoDrop 1000 (Thermo Scientific). The 260/280 and 260/230 ratios were also considered to assess sample purity. The cDNA synthesis was performed using ~500 ng of column‐purified RNA using iScript (Bio‐Rad) following the manufacturer's protocol in a final sample volume of 15 µl. Once prepared, samples were placed into a T100™ Thermal Cycler (Bio‐Rad). The cDNA was generated using the following steps: 25°C for 5 min, 42°C for 30 min, 85°C for 5 min, and 4°C for 5 min. Prior to real‐time PCR, cDNA stocks were diluted 1:20. The no‐reverse transcriptase (NRT) controls were prepared in the same way as above without enzyme using four randomly selected RNA samples.

### 2.6 Real‐time PCR analysis

Primer sets for dopamine transcripts have been reported previously by us (Shontz, Souders, Schmidt, & Martyniuk, 2018). The genes investigated in this study included tyrosine hydroxylase 1 (*th1*), dopamine transporter 1 (*slc6a3*), dopamine receptor D1b (*drd1b*), dopamine receptor D2a (*drd2a*), and dopamine receptor D3 (*drd3*). Superoxide dismutase 1 and 2 (*sod1 and sod2*) were also measured in larval zebrafish. However, both *sod2 and drd3* were too low in expression to confidently measure in the larval pools. Primer sequences are provided in Table S1.

Prior to real‐time PCR, all samples were first diluted 1/20 in DNase–RNase‐free water. Real‐time PCR was performed using the CFX Connect™ Real‐Time PCR Detection System (Bio‐Rad) with SsoFast™ EvaGreen® Supermix (Bio‐Rad), 200–300 nM of each forward and reverse primer, and 3.33 µl of diluted cDNA. The two‐step thermal cycling parameters were as follows: initial 1‐cycle Taq polymerase activation at 95°C for 30 s, followed by 95°C for 5 s, and primer annealing for 5 s (temperature specified in Table S1). After 40 cycles, a dissociation curve was generated, starting at 65.0 and ending at 95.0°C, with increments of 0.5°C every 5 s.

Transcripts of interest were normalized to the geometric mean of two reference genes (actin, cytoplasmic 1 or actb, and rps18). This results in a normalized expression value for every sample. The target stability function in the CFX Manager software determined that the combined M‐value for actb and rps18 was 2.2 (CV = 0.94). Primer sets were tested for linearity and efficiency using a 4‐point standard curve generated by a dilution series from a cDNA pool of embryo samples. The qPCR analysis included three NRT samples and one NTC sample. Negative controls indicated that RNA column purification and DNase treatment sufficiently removed gDNA. Sample sizes were as follows: 0.1% DMSO (*N* = 8), 1 μM pyrovalerone + 0.1% DMSO (*N* = 10), and 10 μM pyrovalerone + 0.1% DMSO (*N* = 9). All primers used in the qPCR analysis amplified one product, indicated by a single melt curve.

### 2.7 Statistical analysis

Oxygen consumption rate (pmol/min/embryos) data were collected with the Wave software (Agilent V2.6). For oxygen consumption rates, an ordinary one‐way ANOVA along with a Tukey's multiple comparisons test was used to test for differences among treatments. Calculations for mitochondrial endpoints were conducted as follows: Basal respiration [defined as mean basal OCR measurement – nonmitochondrial respiration], oligomycin‐induced ATP‐linked respiration [defined as mean basal OCR – mean OCR following oligomycin injection], FCCP‐induced maximum respiration [mean maximum OCR measurement − final NaN3 OCR measurement], spare capacity [difference between maximum respiration and (basal respiration – nonmitochondrial respiration)], proton leak [defined as difference between basal respiration and oligomycin‐induced ATP‐linked respiration], and nonmitochondrial respiration [defined as final plateaued NaN3 OCR] were calculated as per Seahorse XF Cell Mito Stress Test Kit User Guide (User Guide Kit 103015‐100; Agilent).

For the VMR test, an ordinary ANOVA followed by a Holm–Sidak's multiple comparisons test was used to test for differences among experimental groups (*α* = 0.05). If there were differences detected among groups, each time unit (light or dark) was analyzed further for differences within each 10‐min block of time. Units are expressed as total distance moved (mm). For the light–dark preference test, a one‐way ANOVA was used to test for differences among treatments for total velocity in light versus dark (mm/s) followed by a Holm–Sidak's multiple comparisons test (*α* = 0.05) to the vehicle control. A two‐way ANOVA (time and dose) was used to assess mean time in dark zone (average time/visits (seconds)), frequency in dark zone (average number of visits), and cumulative duration in dark zone (time spent as a percentage) followed by a Holm–Sidak's multiple comparisons test. Behavioral profiles were generated in EthoVision software.

Normalized gene expression was determined using CFX Manager™ software with the relative ΔΔCq method (baseline subtracted) (Pfaffl, 2001). Gene expression data were analyzed using an ANOVA followed by a Dunnett's post hoc test for multiple comparisons to the control group. Alpha was set at 0.05, and *p* ≤ .05 indicated a difference among groups. All statistical analyses were performed in Prism (v. 6.0).

## 3 RESULTS

### 3.1 Pyrovalerone toxicity to zebrafish

Pyrovalerone did not cause mortality in the 3‐ or 24‐hr experiments for either embryo (bioenergetics assay) or larvae (behavior assays) experiments. However, for the 100 µM pyrovalerone treatment at 24 hr, there were seizure‐like behaviors in the larval fish. No gross deformities were noted in any embryo or larvae exposed to pyrovalerone for 3 and 24 hr.

### 3.2 Oxygen consumption rates

**Figure 1:**
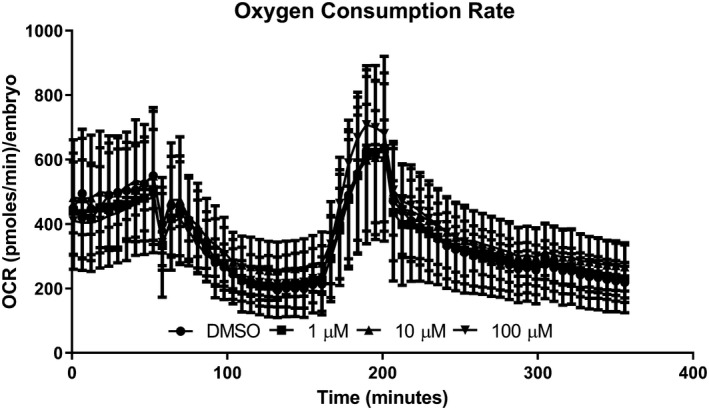
Oxygen consumption rates (pmol min^−1^ embryo^−1^) in zebrafish embryos exposed to pyrovalerone for 24 hr. Each point represents a mean value ± *SD* of the mean (*N* = 5)

The effects of pyrovalerone on mitochondrial bioenergetics in zebrafish embryos were assessed following a 24‐hr exposure to 1, 10, or 100 µM pyrovalerone (Figure 1). The only difference detected was between the 10 and the 100 µM for spare capacity (*F*_(3, 16)_ = 4.51, *p* = .018) (Figure S1D). No differences in OCR were detected for basal respiration (*F*_(3, 16)_ = 0.23, *p* = .88), oligomycin‐induced ATP‐linked respiration (*F*_(3, 16)_ = 0.42, *p* = .74), FCCP‐induced maximum respiration (*F*_(3, 16)_ = 0.51, *p* = .68), proton leak (*F*_(3, 16)_ = 1.12, *p* = .37), and non‐mitochondrial respiration (*F*_(3, 16)_ = 0.40, *p* = .75) (Figure S1A).

### 3.3 Behavioral assessments: locomotor activity and anxiety

**Figure 2:**
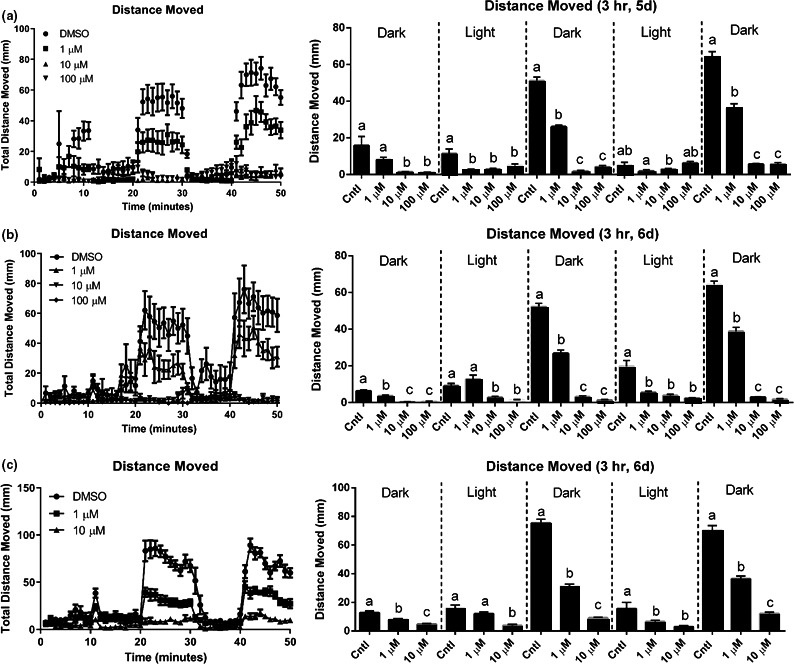
Locomotor analysis of distance moved over the 50 min during a visual motor response (VMR) test after 3‐hr treatment to pyrovalerone. Each graph (left and right) represents an independent experiment. Group mean of the distance‐moved (mm)‐per‐minute intervals for fish (right panel). Total distance moved in each interval of the light and dark. Data are presented as mean value ± *SE*. Sample sizes for each experiment are reported in the methods. Different letters denote significant differences among groups within an interval (*p* < .05)

**Figure 3:**
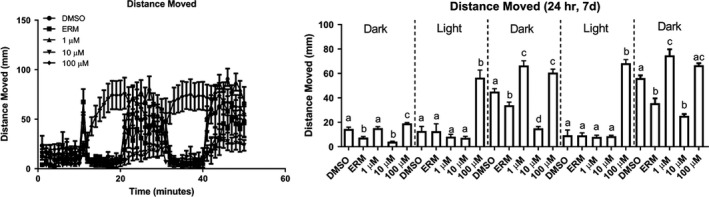
Locomotor analysis of distance moved over the 50 min during a visual motor response (VMR) test after 24‐hr treatment to pyrovalerone. Group mean of the distance‐moved (mm)‐per‐minute intervals for fish (right panel). Total distance moved in each interval of the light and dark. Data are presented as mean value ± *SE*. Sample size is reported in the methods. Different letters denote significant differences among groups within an interval (*p* < .05)

Pyrovalerone treatment induced changes in locomotor activity in zebrafish larvae during the VMR test. Four independent trials were conducted (Figures 2 and 3). In Experiment 1 (3‐hr treatment, 5 dpf) (Figure 2a), there was a significant dose response over time (*F*_(19, 177)_ = 128.4, *p* < .0001), and pyrovalerone decreased distance moved in the dark period compared to light intervals. All doses decreased the distance traveled compared to the control group in the dark period. There was no difference in activity between 10 and 100 µM. In Experiment 2 (3‐hr treatment, 6 dpf) (Figure 2b), pyrovalerone again affected locomotion (*F*_(24, 225)_ = 163.5, *p* < .0001). The response was very similar to that observed in the 5 dpf larval fish, confirming that treatment with pyrovalerone decreases activity at >1 µM. This decrease in activity was dramatic, going from a 50% reduction with 1 µM to a 90% reduction with 10 and 100 µM. In this experiment (3‐hr treatment, 6 dpf) (Figure 2c), a similar dose response was observed as with the previous two experiments (*F*_(16, 153)_ = 127.4, *p* < .0001). Thus, we are confident that pyrovalerone induces hypoactivity in larval zebrafish after a 3‐hr exposure. The final experiment was conducted to determine whether these responses were still evident with longer treatment. Figure 3 shows larval fish at 7 dpf, but following a 24‐hr treatment (*F*_(24, 225)_ = 65.31, *p* < .0001). Responses revealed hyperactivity and higher movement in the treated fish compared to the controls for both 1 and 100 µM. This is likely due to an increase in seizing activity of the fish in the 100 µM treatment.

We also set out to assess anxiolytic/anti‐anxiolytic behaviors in the zebrafish larvae following treatment with pyrovalerone using the light–dark preference test. In the first experiment (3‐hr exposure, 5 dpf), we detected differences among groups for both time and dose. Exposure to pyrovalerone decreased the mean time larvae spent in the dark zone (15‐min bins) (Interaction *F*_(9, 229)_ = 0.661, *p* = .74; Time *F*_(3, 229)_ = 137.4, *p* < .0001; Dose *F*_(3, 229)_ = 3.9, *p* = .010) (Figures S2–S5). Moreover, the frequency within the Dark zone (15‐min bins) was also significantly different between groups (Interaction *F*_(9, 368)_ = 2.67, *p* = .0052; Time *F*_(3, 368)_ = 49.80, *p* < .0001; Dose *F*_(3, 368)_ = 8.87, *p* < .0001). Pyrovalerone decreased the amount of time spent in the dark zone. However, an important point to make is that these responses were observed at the end of the assay (last 15 min). In the second experiment (3‐hr treatment, 5 dpf), latency to first entry into dark zone was different compared to the control (*F*_(3, 80)_ = 3.4, *p* = .0215). Control fish took a longer time to enter the dark zone compared to fish treated with pyrovalerone (1–100 nM). When distance moved was binned into 15‐min intervals in the light–dark preference assay, there was a significant effect of both time and dose (Interaction *F*_(9, 320)_ = 0.84, *p* = .58; Time *F*_(3, 320)_ = 3.10, *p* = .027; Dose *F*_(3, 320)_ = 2.910, *p* = .035). In the last 15 min of the assay, fish moved less than the control fish at doses in the nM range, supporting the experiments above which revealed hypoactivity with pyrovalerone. The frequency of fish moving into the dark zone (15‐min bins) was also affected by dose but not time (Interaction *F*_(9, 320)_ = 0.89, *p* = .55; Time *F*_(3, 320)_ = 0.24, *p* = .87; Dose *F*_(3, 320)_ = 2.82, *p* = .039). Fish treated with pyrovalerone tended to visit the dark zone more frequently than those treated with the solvent control. However, this effect of dose was only observed in the initial 15 min of the assay, and this response was no longer noted after an hour. The same response was noted for “Cumulative Duration in Dark zone” (15‐min bins), and fish treated with pyrovalerone spent more time in the dark zone compared to the controls (Interaction *F*_(3, 320)_ = 1.70, *p* = .77; Time *F*_(3, 320)_ = 1.75, *p* = .14; Dose *F*_(3, 320)_ = 2.94, *p* = .020).

We also tested whether longer treatments with pyrovalerone would induce changes in behaviors related to anxiolytic/anti‐anxiolytic and treated larvae for 24 hr exposure in –6 dpf larvae. In the first 24‐hr experiment, distance moved (15‐min bins) (Interaction *F*_(9, 344)_ = 0.57, *p* = .83; Time *F*_(3, 344)_ = 2.02, *p* = .11; Dose *F*_(3, 344)_ = 3.25, *p* = .022). Mean time in Dark zone (15‐min bin) (Interaction *F*_(9, 275)_ = 0.63, *p* = .77; Time *F*_(3, 275)_ = 47.60, *p* < .0001; Dose *F*_(3, 275)_ = 1.01, *p* = .38). Frequency in Dark zone (15‐min bin) (Interaction *F* = 2.78, *p* = .16; Time *F* = 23.5, *p* < .0001; Dose *F* = 1.67, *p* = .048). Cumulative Duration in Dark zone (15‐min bin) (Interaction *F* = 1.27, *p* = .86; Time *F* = 3.79, *p* = .0032; Dose *F* = 2.29, *p* = .038). However, there was no difference in any group after multiple comparisons. In the second experiment (24‐hr exposure, 6 dpf), there was a response for distance moved (15‐min bin) with zebrafish (Interaction *F*_(9, 364)_ = 0.15, *p* = .99; Time *F*_(3, 364)_ = 13.7, *p* < .0001, Dose *F*_(3, 364)_ = 2.98, *p* = .031); however, there was no difference in any group after a post hoc correction. There were also differences based on the two‐way ANOVA for Frequency in Dark zone (15‐min bin), but only for time and not for dose (Interaction *F*_(9, 364)_ = 0.24, *p* = .99; Time *F*_(3, 364)_ = 45.21, *p* < .0001; Dose *F*_(3, 364)_ = 1.28, *p* = .28). Lastly, Cumulative Duration in Dark zone (15‐min bin) (Interaction *F* = 1.11, *p* = .89; Time *F* = 1.72, *p* = .083; Dose *F* = 4.29, *p* = .0009), but there were no groups different following a post hoc test.

To summarize, unlike the variable of time in the assay, there were no consistent effects of pyrovalerone on any behavioral endpoint measured in the light–dark preference test for both the 3‐ and 24‐hr exposures.

### 3.4 Expression of transcripts in dopaminergic and oxidative damage response

**Figure 4:**
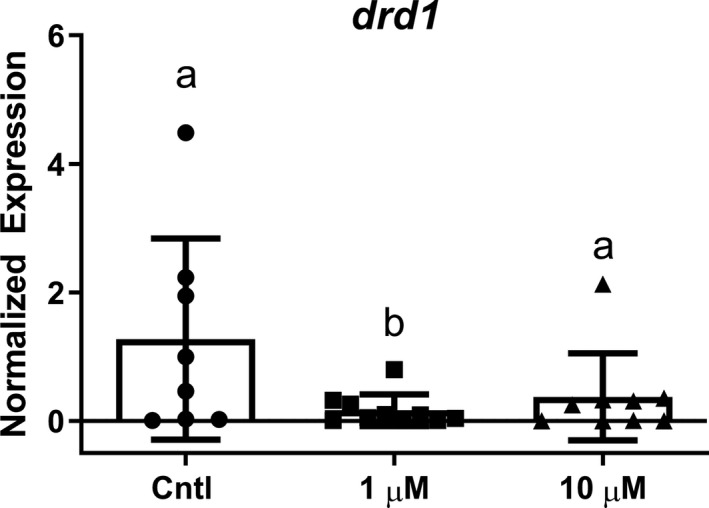
The expression levels of *dat1* mRNA. Data are presented as mean value ± standard error (*N* = 8–10). Different letters reflect a significant difference between the groups (*p* ≤ .05)

Pyrovalerone decreased the expression levels of *drd1b* in zebrafish at 1 µM (*F*_(2, 24)_ = 3.32, *p* = .049), but did not affect the expression levels significantly at 10 µM (Figure 4). There was a trend toward reduced expression in fish exposed to 10 µM pyrovalerone. Pyrovalerone did not affect transcript levels of *th1* (*F*_(2, 24)_ = 0.98, *p* = .39), *slc6a3* (*dat1*) (*F*_(2, 11)_ = 1.40, *p* = .29), or *drd2a* (*F*_(2, 10)_ = 2.20, *p* = .16) (Figure S6).

Since pyrovalerone affected mitochondrial bioenergetics, relative mRNA levels of *sod1* and *sod2* were measured to determine whether there was evidence for oxidative damage response in the embryos (Figure S6). There was no significant difference in transcript levels for *sod1* (*F*_(2, 24)_ = 1.18, *p* = .33) in larvae treated with 1 and 10 µM compared to the control group. Sod2 was too low in expression for many samples and could not be reliably quantified.

## 4 DISCUSSION

The objective of this study was to determine the effects of the cathinone derivative pyrovalerone on mitochondrial bioenergetics, in addition to assessing locomotor and anxiolytic behavioral responses in zebrafish. As cathinones induce hyperactivity and increase metabolic rate (Shortall et al., 2013; Zawilska & Wojcieszak, 2013), we measured oxygen respiration in zebrafish to determine whether there were changes in mitochondrial bioenergetics in intact animals. The rationale was to investigate the potential for pyrovalerone to induce mitochondrial dysfunction in embryos, as early life stages are sensitive to pharmacological and chemical perturbations (Wang, Souders, Zhao, & Martyniuk, 2018a; Zhang, Laurence Souders, Denslow, & Martyniuk, 2017). However, following a 24‐hr treatment with pyrovalerone, there were no detectable effects on respiration at any dose tested. There was also no difference in the expression of *sod1*, a biomarker for oxidative stress response. This suggests that embryos were not under significant oxidative stress with pyrovalerone.

In contrast, other studies point to a role for cathinones in mitochondrial dysfunction, as well as oxidative stress and reactive oxygen species (ROS) formation. A recent study measured the effects of 3‐fluoromethcathinone (3‐FMC), a cathinone derivative, on the oxidative stress response in HT22 mouse hippocampal cells (Siedlecka‐Kroplewska, Wrońska, Stasiłojć, & Kmieć, 2018). The study reported that HT22 cells treated with ~2 mM 3‐FMC for 45 min showed increased ROS compared to the control. In another study, rat liver cells exposed to MDPV showed oxidative damage, impaired mitochondrial activity, depleted ATP stores, and dysregulation of calcium (2+) homeostasis (Valente et al., 2016). Mitochondrial membrane potential dissipation and depleted of ATP levels have been observed in human dopaminergic SH‐SY5Y cells after the introduction of MDMA (Rosas‐Hernandez et al., 2016). Moreover, Luethi and colleagues (Luethi, Liechti, & Krahenbuhl, 2017) quantified the toxic effects of common synthetic cathinones in hepatocytes and showed that MDPV could inhibit complexes I and II of the electron transport chain, thereby reducing mitochondrial membrane potential and acting to deplete ATP. The difference between the aforementioned studies and this study may be related to the model used (cell vs. rat vs. zebrafish), the timing of the treatment, and/or the type of cathinone. For example, single and repeated doses of mephedrone induced significant DNA damage based on the comet assay in adult rats (Kaminska et al., 2019). In our study, zebrafish embryos may have recovered from any oxidative stress after 24 hr. Moreover, although the doses for pyrovalerone used here were effective at modifying behavior, perhaps they did not reach a level that would cause mitochondrial damage.

Pyrovalerone affected locomotor activity in zebrafish following a 3‐hr treatment to >1 µM pyrovalerone in the VMR test (inducing hypoactivity). Conversely, a 24‐hr experiment with pyrovalerone increased the locomotor activity in two of the three doses tested, in both light and dark periods. These responses were determined to be seizure‐like responses in the highest dose of pyrovalerone tested. There were no differences detected between the control and treated fish for the light–dark preference assay, a test designed to evaluate anxiolytic and anti‐anxiolytic behaviors. Both clutch and strain can influence behavior (Baker, Goodman, Santo, & Wong, 2018; Lange et al., 2013), and perhaps, because our animals are from different clutches, there are behavioral differences in the anxiolytic assay due to genetics. Thus, we conclude that unlike locomotor behavior, there is no discernible effect on anxiolytic endpoints based on the assay used.

Other neuroactive pharmaceuticals and drugs of abuse have been investigated using zebrafish behavioral assays. A study conducted by López‐Patiño et al. (2008) studied the relationship between cocaine withdrawal and locomotion in adult male zebrafish (*Danio rerio*, AB wild‐type strain) in a behavioral assay. Cocaine acts, in part, to block the action of the dopamine transporter, serving as a dopamine reuptake inhibitor. After a 72‐hr period of withdrawal from cocaine, adult zebrafish exhibited an increase in basal locomotor activity. However, the researchers found that the administration of varying doses of cocaine hydrochloride (0.015–150 µM) for 75 min to fish undergoing cocaine withdrawal actually counteracted the hyperactive locomotor activity. Thus, prolonged or repeated exposure to dopamine transporter modulators may result in locomotor deficits and loss of activity. In another study conducted by Kyzar et al. (2013), two opposing brain modulators, d‐amphetamine and reserpine, were studied in zebrafish (*Danio rerio*). D‐amphetamine exerts its neurological effects in the brain in part by depleting the abundance of monoamine neurotransmitters such as dopamine, serotonin, and norepinephrine in addition to reversing the transport activity of vesicular monoamine transporter. Adult zebrafish exposed to d‐amphetamine (5 and 10 mg/L) and reserpine (20 and 40 mg/L) for 20 min and up to 7 days produced acute, or immediate, anxiogenic symptoms as well as increased locomotor activity (Kyzar et al., 2013); however, these effects were not observed after 7 days. Our data suggest that pyrovalerone may induce general toxicity and hypoactivity; thus, we decided to explore further a possible mechanism for the loss of activity in larval fish.

Synthetic cathinones inhibit transporters of monoamine neurotransmitters (e.g., dopamine, serotonin, and norepinephrine (Simmler et al., 2013; Simmler, Rickli, Hoener, & Liechti, 2014)). As such, we investigated the dopamine system as a potential mechanism for cathinone‐induced changes in locomotor behavior. There was a downregulation of the dopamine D1 receptor *drd1b* following pyrovalerone treatment, but there was no change in any other transcript investigated. Downregulation of postsynaptic dopamine receptors may act as a compensatory response to prolonged DAT inhibition and excessive dopamine. These data are consistent with other studies that investigate drugs of abuse that affect the DAT. Ashok, Mizuno, Volkow, and Howes (2017) conducted a meta‐analysis to detect associations between stimulants (cocaine, amphetamines, and methamphetamines) and alterations to the dopaminergic system. The researchers reported that there was a reduction in dopamine D2 and D3 receptors (proteins) in users of cocaine and amphetamine‐like stimulants; dopamine D1 receptors, however, were not discussed in this meta‐analysis. Mendez et al. (2001) found that chronic amphetamine administration decreased dopamine D2 receptor expression in the caudate–putamen and in the lateral habenular nucleus of rats, two brain regions integral to the dorsal diencephalic conduction system. In another study, Chiang, Chen, and Chen (2003) treated rats with 5 mg/kg amphetamine for seven days and observed that protein levels for dopamine D3 receptors were downregulated in the limbic forebrain; the researchers also reported that mRNA levels were reduced in amphetamine‐treated animals. Thus, there is evidence that DAT‐acting drugs can regulate dopamine receptor expression. Conversely, the administration of cocaine has been shown to increase initial levels of D1 receptor proteins as reported by Tobón, Catuzzi, Cote, Sonaike, and Kuzhikandathil (2015). However, it is plausible that chronic exposure to amphetamines, and cathinones, could reduce D1 receptor expression after an acute increase in D1 proteins. Nevertheless, we demonstrate that acute exposure to pyrovalerone can suppress the expression of select dopamine receptor isoforms in zebrafish.

Studies describe a close relationship between the dopamine system and larval zebrafish behavior, and there may be a direct link between *drd1* and locomotor activity. Irons and colleagues (Irons, Kelly, Hunter, Macphail, & Padilla, 2013) investigated the functional relationship between dopamine and locomotion by exposing wild‐type larval zebrafish at 6 dpf to nonlethal (0.2–50 µM) doses of dopamine receptor agonists and antagonists; behavioral assays were subsequently conducted to assess locomotor activity. The compounds used included two selective dopamine receptor agonists, SKF‐38393 (selective D₁/D₅ receptor partial agonist) and quinpirole (D₂ and D₃ receptor agonist), as well as two selective dopamine receptor antagonists, SCH‐23390 (D₁ receptor antagonist) and haloperidol (multiple receptors). A nonselective dopamine agonist (apomorphine) and a nonselective dopamine antagonist (butaclamol) were also used in experiments. Irons and colleagues (Irons et al., 2013) found that all drugs used in the study modulated locomotor activity in a dose‐dependent manner; SKF‐38393 and quinpirole increased larval activity, while SCH‐23390 and haloperidol decreased larval activity. Similar to the selective agonists, apomorphine increased activity at all doses. Butaclamol, however, increased activity at low‐to‐medium doses and decreased activity at high doses. In another study, domperidone (DMP), a D2 receptor antagonist, was used by Shontz et al. (2018) to assess the role of DMP in dopaminergic signaling and behavior by exposing 48 hpf  zebrafish (*Danio rerio*, ABTu strain) to varying doses of DMP (1 and 10 µM) for a period of 24 hr; the researchers subsequently measured the locomotor activity and relative expression levels of dopaminergic transcripts (i.e., receptors and transporters) in the treated zebrafish. Locomotor activity was assessed by distance traveled in a behavioral assay using alternating periods of light and dark, and gene expression was measured via qPCR analysis. The study showed that (a) DMP upregulated dopamine receptor transcripts (*drd1*, *drd7*, *drd4b*, and *drd4c*); (b) DMP upregulated dopamine active transporter; and (c) DMP induced hyperactivity. Thus, there is evidence for associations between dopamine receptor expression and larval activity.

In summary, we demonstrate that behavioral screening using larval zebrafish may be a useful screening approach for bath salt derivatives and, coupled with molecular endpoints, may reveal novel insight into the neural mechanisms underlying drug abuse associated with bath salts. Behavioral fingerprinting may be useful to predict adverse outcomes for emerging synthetic drugs, as new moieties enter the illegal drug market. Future work will continue to assess molecular and behavioral responses in alternative animal models, moving toward functional high‐throughput screening for a diverse class of cathinones. Future experiments should focus more on the relationship between cathinones and dopamine receptor signaling in zebrafish as drd1 and dopamine could be the link between behaviors induced by bath salts. Indeed, studies in mice reveal that both methcathinone and 3‐fluoromethcathinone increase dopamine and stimulate spontaneous horizontal locomotor activity in mice, whereas the selective DA receptor D1 antagonist SCH 23,390 blocks the response (Wojcieszak, Andrzejczak, Wojtas, Golembiowska, & Zawilska, 2019). Additional studies are expected to shed light on dose–exposure relationships and relative toxicity of cathinones to vertebrates.

## CONFLICT OF INTEREST

There are no conflicts of interest to declare.

## Data Availability

The data that support the findings of this study are available from the corresponding author upon reasonable request.
