# Corrigendum

**DOI:** 10.1002/brb3.2123

**Published:** 2021-06-17

In the article by Souders et al., the second author name was listed incorrectly. The correct name is Hunter B. Davis.
