# Supplementary material for: The psychoactive cathinone derivative pyrovalerone alters locomotor activity and decreases dopamine receptor expression in zebrafish (Danio rerio)
Source: Brain Behav. 2019 Oct 18;9(11):e01420. doi: 10.1002/brb3.1420 (PMC6851804; doi:10.1002/brb3.1420)
Supplement: Supplementary file 1 [file BRB3-9-e01420-s001.docx]

**Supplemental Table 1.** Primer sets used to amplify genes following treatment to pyrovalerone. Table S1. Primers used for real-time PCR analysis. Provided in the table are the annealing temperature, NCBI accession numbers when available. T_m_ = Annealing Temperature (adapted from Wang et al., 2018).

| **Symbol** | **Gene Name** | **Forward Primer (5’ to 3’)** | **Reverse Primer (5’ to 3’)** | **T_m_ (°C)** | **NCBI accession #** | |
| --- | --- | --- | --- | --- | --- | --- |
| *Housekeeping* | | | | | |  |
| *^a^β-actin* | beta-actin | CGAGCAGGAGATGGGAACC | CAACGGAAACGCTCATTGC | 58 | AF057040 | |
| *^a^rps18* | ribosomal subunit 18 | TCGCTAGTTGGCATCGTTTATG | CGGAGGTTCGAAGACGATCA | 58 | BX296557 | |
| *Oxidative Stress* | | | | | |  |
| *^c^sod1* | superoxide dismutase 1 | CGTCTATTTCAATCAAGAGGGTG | GATGCAGCCGTTTGTGTTGTC | 58 | NM_131294.1 | |
| *^c^sod2* | superoxide dismutase 2 | CTTGGGATAGATGTCTGGG | GTGGTCTGATTAATTGTGCG | 58 | AY195857.1 | |
| *Dopaminergic System* | | | | | |  |
| *^g^th* | tyrosine hydroxylase 1 | GACGGAAGATGATCGGAGACA | CCGCCATGTTCCGATTTCT | 58 | NM_131149.1 | |
| *^h^dat* | dopamine active transporter | AGACATCTGGGAAGGTGGTG | ACCTGAGCATCATACAGGCG | 58 | NM_131755.1 | |
| *^h^drd1* | dopamine receptor d1 | ACGCTGTCCATCCTTATCTC | TGTCCGATTAAGGCTGGAG | 58 | NM_001135976.2 | |
| *^h^drd2a* | dopamine receptor d2a | TGGTACTCCGGAAAAGACG | ATCGGGATGGGTGCATTTC | 58 | NM_183068.1 | |
| *^h^drd3* | dopamine receptor d3 | ATCAGTATCGACAGGTATACAGC | CCAAACAGTAGAGGGCAGG | 58 | NM_183067.1 | |

Wang XH, Zheng SS, Huang T, Su LM, Zhao YH, Souders CL 2nd, Martyniuk CJ. Fluazinam impairs oxidative phosphorylation and induces hyper/hypo-activity in a dose specific manner in zebrafish larvae. Chemosphere. 2018 Nov;210:633-644. doi: 10.1016/j.chemosphere.2018.07.056. Epub 2018 Jul 12. PubMed PMID: 30031347.
