# Supplementary material for: The psychoactive cathinone derivative pyrovalerone alters locomotor activity and decreases dopamine receptor expression in zebrafish (Danio rerio)
Source: Brain Behav. 2019 Oct 18;9(11):e01420. doi: 10.1002/brb3.1420 (PMC6851804; doi:10.1002/brb3.1420)
Supplement: Supplementary file 2 [file BRB3-9-e01420-s002.docx]

**Supplemental Figures**





**Supplemental Figure 1.** (A) Basal Respiration, (B) Oligomycin-Induced ATP-Linked Respiration, (C) FCCP-induced Maximal Respiration (D) Spare Capacity, (E) Proton Leak, and (F) Non-Mitochondrial Respiration. Columns represent mean ± SEM (P < 0.05, one-way ANOVA). Different letters represent differences between groups.





**Supplemental Figure 2.** Light-dark preference test for zebrafish treated with pyrovalerone for 3 hours starting at 5 dpf. (A) Total Activity (B) Total Velocity, (C) Latency to First Visit in the Dark Zone (D) Velocity in Light (left) and Dark (right); (E) Distance Moved; (F) Mean Time in Dark Zone; (G) Frequency in Dark Zone; (H) Cumulative Duration in Dark Zone. Points on the line represent mean ± SEM (P < 0.05, two-way ANOVA).





**Supplemental Figure 3.** Light-dark preference test for zebrafish treated with pyrovalerone for 3 hours starting at 5 dpf. (A) Total Activity (B) Total Velocity, (C) Latency to First Visit in the Dark Zone (D) Velocity in Light (left) and Dark (right); (E) Distance Moved; (F) Mean Time in Dark Zone; (G) Frequency in Dark Zone; (H) Cumulative Duration in Dark Zone. Points on the line represent mean ± SEM (P < 0.05, two-way ANOVA).





**Supplemental Figure 4.** Light-dark preference test for zebrafish treated with pyrovalerone for 24 hours starting at 5 dpf. (A) Total Activity (B) Total Velocity, (C) Latency to First Visit in the Dark Zone (D) Velocity in Light (left) and Dark (right); (E) Distance Moved; (F) Mean Time in Dark Zone; (G) Frequency in Dark Zone; (H) Cumulative Duration in Dark Zone. Points on the line represent mean ± SEM (P < 0.05, two-way ANOVA).





**Supplemental Figure 5.** Light-dark preference test for zebrafish treated with pyrovalerone for 24 hours starting at 5 dpf. (A) Total Activity (B) Total Velocity, (C) Latency to First Visit in the Dark Zone (D) Velocity in Light (left) and Dark (right); (E) Distance Moved; (F) Mean Time in Dark Zone; (G) Frequency in Dark Zone; (H) Cumulative Duration in Dark Zone. Points on the line represent mean ± SEM (P < 0.05, two-way ANOVA).





**Supplemental Figure 6.** The expression levels of (A) tyrosine hydroxylase 1 (*th*), (B) dopamine active transporter 1 (*dat*), (C) superoxide dismutase 1 (*sod1*) and (D) dopamine receptor 2a (*drd2a*) in 0.1% DMSO, 1 or 10 μM pyrovalerone at 144. Data are presented as mean value ± standard error (N = 6-7). Asterisks (*) indicate a significant difference between the treatment and the control at p < 0.05.
